# Nitrogen-related intermediate band in P-rich GaN_x_P_y_As_1−x−y_ alloys

**DOI:** 10.1038/s41598-017-15933-1

**Published:** 2017-11-16

**Authors:** K. Zelazna, M. Gladysiewicz, M. P. Polak, S. Almosni, A. Létoublon, C. Cornet, O. Durand, W. Walukiewicz, R. Kudrawiec

**Affiliations:** 1 Faculty of Fundamental Problems of Technology, Wroclaw University of Science and Technology, Wybrzeze Wyspianskiego 27, 50-370 Wroclaw, Poland; 2 UMR FOTON, CNRS, INSA-Rennes, F-35708 Rennes, France; 3 Materials Sciences Division, Lawrence Berkeley National Laboratory, Berkeley, California 94720 USA

## Abstract

The electronic band structure of phosphorus-rich GaN_x_P_y_As_1−x−y_ alloys (x ~ 0.025 and y ≥ 0.6) is studied experimentally using optical absorption, photomodulated transmission, contactless electroreflectance, and photoluminescence. It is shown that incorporation of a few percent of N atoms has a drastic effect on the electronic structure of the alloys. The change of the electronic band structure is very well described by the band anticrossing (BAC) model in which localized nitrogen states interact with the extended states of the conduction band of GaAsP host. The BAC interaction results in the formation of a narrow intermediate band (E_−_ band in BAC model) with the minimum at the Γ point of the Brillouin zone resulting in a change of the nature of the fundamental band gap from indirect to direct. The splitting of the conduction band by the BAC interaction is further confirmed by a direct observation of the optical transitions to the E_+_ band using contactless electroreflectance spectroscopy.

## Introduction

The concept of intermediate band solar cells (IBSCs) has been originally proposed by Wolf^1^. Later Luque and Martí^2^ have shown that an IBSC with properly located bands could attain very high solar power conversion efficiencies. The elegant simplicity of the IBSC concept has motivated researchers to search for novel materials and materials structures with an intermediate band that would satisfy strict requirements regarding photon absorption and charge collection^3–5^. In general, the intermediate band materials/structures can be divided into three main groups: nanostructures, such as quantum dots (QDs)^4–7^; semiconductor bulk materials containing a high density of deep-level impurities^4,5,8^; and highly mismatched alloys (HMAs)^9–11^. An important example of intermediate band HMA is the GaNPAs alloy in which P to As ratio can be tuned at will to change the band gap and modify the respective offsets between the conduction band edge and the localized N level energy making this alloy one of the most promising materials for IBSC applications^11–14^. The possibility of growing GaNPAs on Si substrates is a very important advantage of this alloy as it makes it feasible to co-integrate IBSC, multi-junctions solar cells^15^ or laser emitters with Si technology. For these reasons As-rich GaNPAs alloys and quantum wells with a few percent of nitrogen atoms have been investigated very intensively in recent years^16,17^. An electrically pumped laser on Si substrate with the active region containing GaNPAs quantum wells has been demonstrated by Liebich *et al*.^18^. Recently GaN_x_P_y_As_1−x−y_ alloy with y ~ 0.4 and a few percent of nitrogen has been identified and studied as the optimal material for the IBSC applications^11,13,19^. Much less research has been done on the GaN_x_P_y_As_1−x−y_ alloys with P concentration larger than y ≥ 0.6^20–22^. Especially, although this composition range is interesting from the viewpoint of the intermediate band formation and the lattice matching with Si substrates in a multi-junction approach, complementary experimental and theoretical investigations of the whole conduction band properties was not proposed yet^23^.

**Figure 1:**
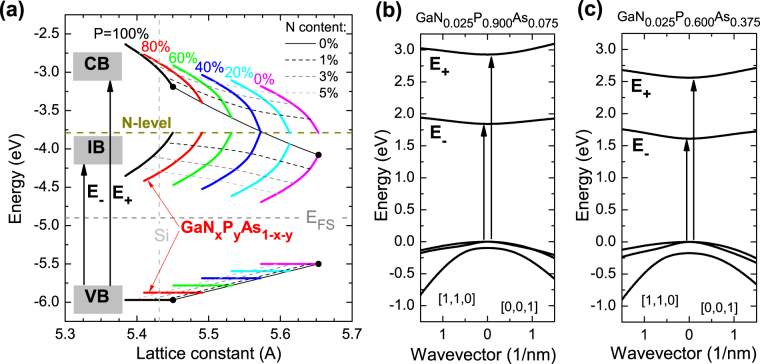
(**a**) Energies of VB, IB (E_−_), and CB (E_+_) band edges versus the lattice constant in unstrained bulk GaN_x_P_y_As_1−x−y_ with various P concentrations (thick color lines). Dashed lines correspond to GaN_x_P_y_As_1−x−y_with the same nitrogen concentration: x = 0.01 – dark grey lines; x = 0.03 – grey lines; x = 0.05 – light grey lines. Calculated energies of the E_+_ and E_−_ bands relative to the valence band edge for GaN_0.025_P_0.9_As_0.175_ (**b**) and for GaN_0.025_P_0.6_As_0.375_ (**c**). These calculations have been performed according to the BAC model described in the Methods section and ref.^11^.

As seen in Fig. 1(a) the energy level of single nitrogen atoms is located below the conduction band of GaP_y_As_1−y_ host for the y > 0.4 compositions of the unstrained alloy. According to the BAC model the interaction of the localized N level with the states of the GaPAs host leads to formation of E_−_ and E_+_ bands in GaNPAs alloy^11^, see mathematical formulas for E_−_ and E_+_ bands in the Methods section. Figure 1(b) and (c) show the dispersion relations for E_+_ and E_−_ bands in GaN_0.025_P_0.9_As_0.075_ and GaN_0.025_P_0.6_As_0.375_ calculated according to the model described in the Methods section. As is seen in this case the E_−_ band forms a narrow intermediate band (IB), that is well separated from the E_+_ band i.e., the upper conduction band (CB). These assertions of the BAC model have been never fully confirmed. Specifically, the optical transitions from the valence band (VB) edge to the E_+_ band were not observed yet in P-rich GaNPAs alloys. In this work, we have applied several optical spectroscopy methods including contactless electroreflectance (CER), photomodulated transmission (PT), optical absorption, and photoluminescence (PL) to study the electronic band structure and the changing character of bandgap in P-rich GaNPAs alloys. We show that all the experimental results can be consistently explained by the BAC model and confirm that GaNPAs alloys could be utilized for IBSC applications.

## Results and Discussion

In order to delineate the effects of the GaPAs host matrix composition from the effects of nitrogen we have grown two sets of samples. The first set consists of GaN_x_P_1−x_ layers with various nitrogen concentrations (x = 0.005, 0.013, 0.015, 0.023, and 0.025), and the second set are GaN_x_P_y_As_1−x−y_ layers with x = 0.025 and various P concentrations (y = 0.6, 0.7, 0.9, 0.95). All the samples were grown on GaP substrate by molecular beam epitaxy (MBE). Relevant details of the growth are described in the Methods section.

### Optical properties of GaNP alloy

First, we present the results of the measurements of optical properties on GaNP samples. It is worth noting that although GaNP alloy has been studied quite intensively for last few years^24–29^ there is only a limited number of papers reporting experimental observation of optical transitions to the E_+_ subband^26,27^, and no experimental evidence of the spin-orbit splitting of the valence band of the alloy.

**Figure 2:**
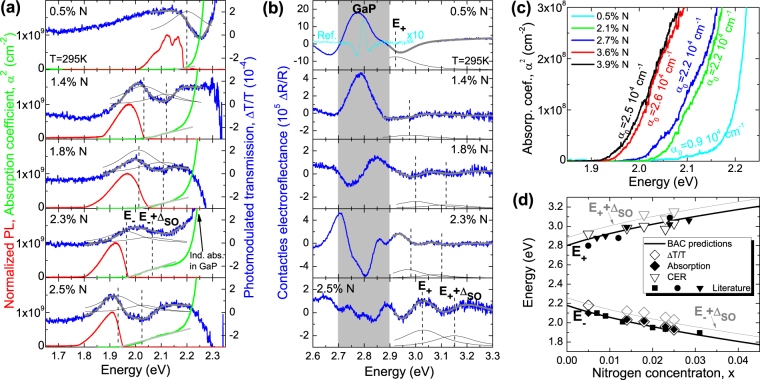
(**a**) Absorption (green lines) and photomodulated transmission (blue lines) spectra of GaN_x_P_1−x_ layers with various N concentrations measured in the vicinity of E_−_ and E_−_ + Δ_SO_ transitions together with low temperature photoluminescence spectra (red line). (**b**) Contactless electroreflectance spectra of GaNP layers (blue lines) measured in the vicinity of E_+_ transition. The fitting curves are shown by thick grey lines. Modulus of individual resonances are shown by thin solid black lines. Since GaNP layers are tensily strained the fundamental transition is between the light-hole band and the E_−_ band. In our fitting the strain induced splitting between light- and heavy-hole subbands is neglected and a single resonance is used to simulate the E_−_ and the E_+_ transition. (**c**) Absorption curve in the vicinity of absorption edge used to determine the absorption constant *α*_0_. (**d**) Comparison of energies of E_−_, E_−_ + Δ_SO_, E_+_, and E_−_ + Δ_SO_ transitions obtained from BAC model for GaNP with various N concentrations (solid lines) with experimental data (points) obtained in this work and taken from the literature^26,27^.

Figure 2(a) shows photomodulated transmission (blue line), photoluminescence (red line) and absorption (green line) spectra (i.e., a square of the absorption coefficient obtained from transmittance and reflectance measurements) for GaNP layers with various N concentrations. The strong absorption, observed for the photon energies above ~2.2 eV, comes from the indirect gap of the thick GaP buffer/substrate. Additional absorption edge is clearly observed at lower energies. The absorption edge shifts to longer wavelengths with increasing nitrogen concentration. The linear slope of the α^2^ plot indicates that the low energy absorption edge originates from direct optical transitions. The direct character of this bandgap is further confirmed by PL and PT measurements.

Low temperature PL spectra presented in Fig. 2(a) clearly show quite significant Stokes shift with the PL peak energy falling significantly below the absorption edge energy. The shift can be attributed to large random fluctuations of the direct bandgap that lead to exciton localization and a shift of the PL peak to lower energy^19^. Additionally, as shown in Fig. 2(a) clear observation of the optical transitions with PT provides further support for the direct nature of the optical transitions^9–11,30,31^. In PT spectra two resonances can be resolved. These resonances are related to optical transitions between the VB edge and the E_−_ band (transition labeled as E_−_) and the spin-orbit split-off band and the E_−_ band (transition labeled as E_−_ + Δ_SO_). In order to determine energies and broadening of these transitions, PT resonances are fitted by Aspnes’ formula^32^

1 $$\frac{{\rm{\Delta }}T}{T}(E)={\rm{Re}}[C{e}^{i\vartheta }{(E-{E}_{j}+i{\rm{\Gamma }})}^{-m}]+f(E),$$

where $\frac{{\rm{\Delta }}T}{T}(E)$ is the energy dependent PT signal, C and ϑ are the amplitude and phase of the resonance, and E_j_ and Γ are the energy and the broadening parameter of the optical transition, respectively, m depends on the type of optical transition and is assumed to be m = 2.5 in this case. $f(E)$ is a parabolic function which simulates the background sign related to Fabry-Perot oscillation^33,34^ or a wing of GaP-related signal. The fitted curves are shown as thick grey lines in Fig. 2(a) together with the moduli of PT resonances, which are shown as solid black lines. The moduli of PT resonances (ρ) are obtained according to Eq.(2) with parameters taken from the fit.

2 $${\rm{\Delta }}\rho (E)=\frac{|C|}{{[{(E-{E}_{j})}^{2}+{{\rm{\Gamma }}}^{2}]}^{\frac{m}{2}}}$$

Comparing the energy of E_−_ transition, which is a direct optical transition in PT, with the energy of absorption edge determined from the α^2^ plot it has been found that they are the same within the range of experimental uncertainties. It confirms the direct gap character of bandgap observed in absorption for this alloy.

The photomodulated transmission cannot be used to observe the higher energy E_+_ transitions in GaNP because of a strong absorption of the GaP substrate. Therefore, the CER has been applied to measure this transition. In this case, besides the E_+_ transition in GaNP, very strong resonances related to E_0_ and E_0_ + Δ_SO_ transitions in GaP buffer layer are also observed. The observation of these resonances indicates a weak light absorption in this spectral range in GaNP layer which confirms intermediate character of E_−_ band in GaNP whose density of states is determined by the N content.

An interesting feature of the CER spectra shown in Fig. 2(b) is the large broadening of GaP-related resonances measured for samples with the top GaNP layer. This broadening is few times larger than the broadening of the same transition in the reference GaP epilayer, see CER spectrum for the reference sample plotted by light blue line in Fig. 2(b). In general an increase of broadening of CER resonance can be attributed to the alloying related bandgap fluctuations and/or a strong built-in electric field. Since the GaP buffer is a binary compound, the large broadening of GaP-related resonance in the studied samples can be only due to a strong band bending at the GaNP/GaP interface.

As shown previously^35,36^, the Fermi level in as-grown GaInNAs(Sb) tends to be pinned at the Fermi stabilization energy (*E*_*FS*_) because of native defects in this material. The same phenomenon is expected for GaNP(As) alloys. In this case the *E*_*FS*_ is located in the middle of the bandgap as shown in Fig. 1. On the other hand the Fermi level in *n*-type GaP is located near the conduction band. This leads to formation of a strong band bending at the GaNP(As)/GaP interface (i.e., an inhomogeneous built-in electric field in these layers). The CER signal originates from modulation of the built-in electric field. In the case of spatially homogeneous field this leads to a CER resonance followed by a Franz-Keldysh oscillation (FKO)^37–39^. However, the FKO can be damped and not observed for an inhomogeneous built-in electric field. In such case presence of a strong inhomogeneous electric field manifests itself in a large broadening of CER resonance as is observed for GaNP samples.

The CER resonances related to E_+_ and E_+_ + Δ_SO_ transitions in GaNP are expected for energies higher than 2.8 eV, i.e. above the direct gap of pure GaP. Therefore it is possible to observe these transitions separately from the transitions originating from the GaP epilayers/substrates. Fits of the experimental curves fitted with Aspnes’ formula^32^ given by Eq. (2) are shown as thick grey lines in Fig. 2(b) together with the moduli of CER resonances, represented by solid black lines.

Figure 2(c) presents the square of the absorption coefficient in the vicinity of absorption edge. These results allow to determine the absorption edge and the absorption constant *α*_0_. Values of *α*_0_ determined for GaNP layers with different N concentrations are given in Fig. 2(c) and discussed in details in the next part of this paper.

Figure 2(d) compares the energies of E_−_, E_−_ + Δ_SO_, E_+_, and E_+_ + Δ_SO_ transitions calculated using the BAC model with the energies of direct optical transitions determined from PT (open diamond points) and CER (open triangle points) measurements as well as the absorption edge energy determined from transmission and reflectance measurements (solid diamond points). In addition, energies of E_−_ and E_+_ transitions taken from the literature^26,27^ are shown in this figure (other solid points). Taking into account the experimental uncertainties the found energies of E_−_, E_−_ + Δ_SO_, E_+_, and E_+_ + Δ_SO_ transitions are in a good agreement with BAC predictions.

### Optical properties of GaNPAs films

**Figure 3:**
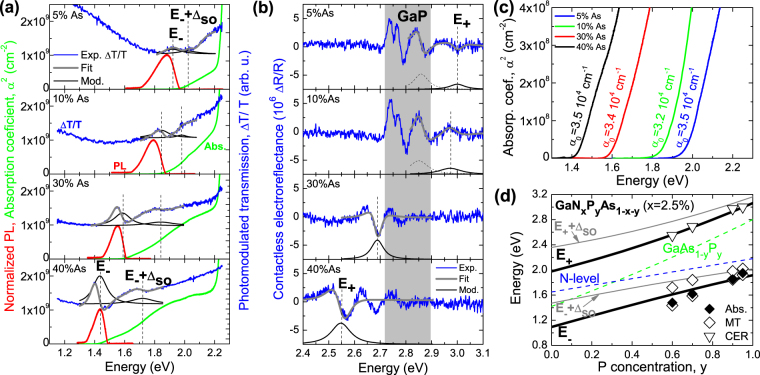
(**a**) Absorption (green lines) and photomodulated transmission (blue lines) spectra of GaN_x_P_y_As_1−x−y_ layers with x = 0.025 and various P concentrations measured in the vicinity of E_−_ and E_−_ + Δ_SO_ transitions together with low temperature photoluminescence spectra (red line). (**b**) Contactless electroreflectance spectra of GaNPAs layers (blue lines) measured in the vicinity of the E_+_ transition. The fitting curves are shown by thick grey lines. Modulus of individual resonances are shown by thin solid black lines. Due to the compressive strain in the studied GaNPAs layers a splitting between light- and heavy-hole subbands is present but this splitting is neglected and a single resonance is used to simulate the E_−_ and the E_+_ transition. This resonance is attributed to the heavy-hole subband as the dominant contributor in this case. (**c**) Absorption curve in the vicinity of absorption edge used to determine the absorption constant *α*_0_. (**d**) Comparison of energies of E_−_, E_−_ + Δ_SO_, E_+_, and E_−_ + Δ_SO_ transitions obtained from BAC model for GaN_x_P_y_As_1−x−y_ with x = 0.025 and various P concentrations (solid lines) with experimental data (points).

The results of previous section show that the BAC model well describes the N dependence of the electronic band structure of GaNP alloy. The current section discusses the effect of BAC interaction in GaNPAs. It is shown in Fig. 1(a) that the energy separation between the nitrogen level and the conduction band of the GaPAs host varies with As concentration. Figure 3(a) shows room temperature absorption (green line), photomodulated transmission (blue line), and low temperature photoluminescence (red line) spectra for GaN_x_P_y_As_1−x−y_ layers with x ~ 0.025 and various P concentrations (y = 0.6, 0.7, 0.9, and 0.95) in the vicinity of E_−_ and E_−_ + Δ_SO_ transitions. The PT spectra are fitted with Aspnes’ formula. The moduli of PT resonances are plotted as thin black lines in Fig. 3(a). Similar as in the case of GaNP layers two resonances are observed in PT spectra. They are attributed to optical transitions between the VB and the E_−_ band (transition labeled as E_−_) and between spin-orbit split-off VB and the E_−_ band (transition labeled as E_−_ + Δ_SO_).

The results presented in Fig. 3 show that similarly as in the case GaNP the P-rich GaNPAs alloy exhibits a direct band gap. This is evidenced by well resolved PT resonances as well as the fundamental absorption edge with linear dependence of α^2^ on the photon energy. This conclusion is further supported by a strong PL observed for these samples, see Fig. 3(a).

The results of CER measurements of GaNPAs films are presented in Fig. 3(b). Notably the E_+_ transition energy in GaN_x_P_y_As_1−x−y_ shifts from about 3 eV for y = 0.95 to 2.55 eV for y = 0.6. CER spectra for these samples in the vicinity of E_+_ transitions are shown in Fig. 3(b). As seen in Fig. 3(b) the optical transitions associated with GaP substrate are observed only in GaN_x_P_y_As_1−x−y_ samples with high P content (y = 0.95 and y = 0.9). These transitions are not observed for the films with lower P content because, as shown in Fig. 3(c) the fundamental band gap absorption associated with transitions between the VB and E_−_ shifts to lower energy preventing the higher energy photons from reaching the GaP substrate. At y = 0.6 and y = 0.7 the E_+_ transition is observed below the direct gap in GaP and therefore the E_0_ transition in GaP is not observed for these samples. For GaN_x_P_y_As_1−x−y_ with y = 0.9 and y = 0.95 the E_+_ transition is located only slightly above the direct gap in GaP. Therefore, CER resonances related to E_+_ and E_+_ + Δ_SO_ transitions in GaNPAs layers almost overlap with direct optical transitions in GaP making a quantitative analysis and fitting of CER spectrum rather difficult. An additional complication comes from the presence of strong built-in electric field in these samples which limits the application of Aspnes’ formula. The CER spectra for the two large P content samples are fitted by contributions from two resonances: one attributed to GaNPAs sample and the other associated with the high energy part of GaP-related signal. GaN_x_P_y_As_1−x−y_ samples with y = 0.6 and 0.7 are fitted by a single resonance. In this case the E_+_ transition does not overlap with the GaP-related transitions, but this transition is followed by FKO, which is weakly damped, and thereby the E_+_ + Δ_SO_ transition is not well resolved in CER spectrum.

Figure 3(c) presents the square of the absorption coefficient in the vicinity of absorption edge. These results, the same as for GaNP, also enable to determine the absorption edge and the absorption constant *α*_0_. In this case values of *α*_0_ are also given in Fig. 3(c) and as seen they are very similar for the four GaNPAs layers with different P concentrations. These values are compared with the literature data in the next part of this paper.

Figure 3(d) shows energies of E_−_, E_−_ + Δ_SO_, E_+_, and E_+_ + Δ_SO_ transitions obtained within the BAC model for GaNPAs alloys with various P concentrations together with experimental data: energies of direct optical transitions determined from PT (open diamond points) and CER (open triangle points) measurements as well as the absorption edge determined from transmission and reflectance measurements (solid diamond points). In this case the comparison of experimental data with the plotted BAC predictions is more complicated, since nitrogen concentration in GaN_x_PyAs_1−x−y_ samples may vary by ±0.5% from sample to sample. In this case, it is rather difficult to control the N concentration (x) at the constant level while varying the P to As ratio. The results in Fig. 3(d) show a reasonably good agreement between the theory and the experiment considering the limited accuracy of the measurements and the data fitting methods.

**Figure 4:**
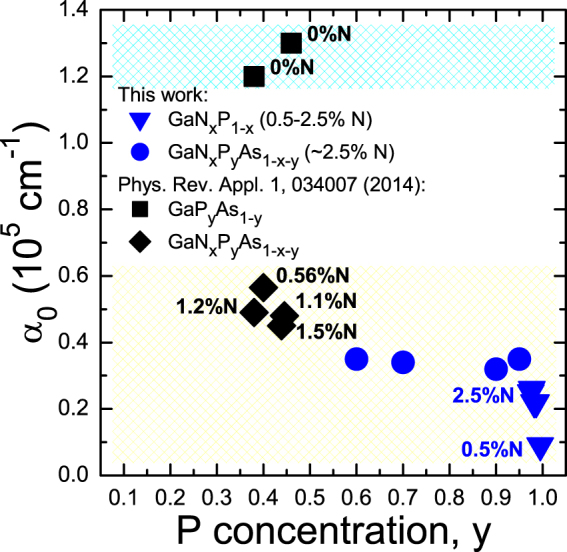
The absorption constant *α*_0_ determined for GaNP (solid blue triangles) and GaNPAs (solid blue circles) layers together with the literature data from ref.^11^.

The results of the optical measurements discussed in the previous sections demonstrate that incorporation of N into the GaPAs host matrix results in formation of an intermediate band whose location relative to the conduction and valence band edges can be controlled by the As to P ratio and by the N content. The key requirement for the IB material suitable for photovoltaic application is a strong optical absorption between the VB and the IB as well as between the IB and the CB. As discussed previously the direct gap character of the absorption edge in GaNPAs alloy is confirmed by the linear slope in the α^2^ plot. The absorption coefficient can be written as follows in Eq.(3)

3 $$\alpha (E)={\alpha }_{0}\sqrt{\frac{E-{E}_{g}}{{E}_{g}}},$$

where *α*_0_ represents the strength of the optical absorption, *E*_*g*_ is the energy gap and *E* is the photon energy. The values of *α*_0_ determined from fitting Eq. 3 to the experimental data in Figs. 2(c) and 3(c) are shown in Fig. 4 together with the previously presented data for GaNPAs samples with lower P concentration^11^. As shown here the currently measured values of *α*_0_ constants are very consistent with the previous data. The results in Fig. 4 show that incorporation of nitrogen into P-rich GaPAs host leads to formation of IB with the absorption coefficient of about 0.2–0.4 × 10^5^ cm^−1^ for VB → IB transition (E_−_ transition). This indicates that the GaNPAs thin films have the optical absorption for VB to IB transitions strong enough to be used in thin film IB solar cells.

It is important to note that the IB→CB transition is also interesting to explore from the viewpoint of application of GaNP(As) alloys in IB solar cells. But for this purpose *n*-type samples are needed since IB should be partially occupied by electrons in order to obtain an absorption between the IB and the CB. The studied samples are nominally undoped with the unintentional electron concentration below 10^17^ cm^−3^ and thereby they are not useful for this study. Therefore a further studies of *n*-type GaNP(As) will be very interesting in this case.

## Summary

It is shown that incorporation of a few percent of nitrogen into P-rich GaPAs leads to formation of intermediate band and the change of the nature of the fundamental band gap from indirect to direct. The direct band gap in GaNPAs alloys has been confirmed by absorption and PL measurements. In addition direct optical transitions between the valence band and the upper conduction band (E_+_ transition) have been clearly identified in optical CER spectra. The experimentally observed N-induced modification of the electronic band structure are well accounted for by the BAC model. The observed formation of the isolated intermediate band offers a potential of using P-rich GaNPAs alloys for intermediate band solar cells.

## Methods

### BAC model

According to the BAC model in GaNP and GaNPAs^11^ the interaction of N-related states with a conduction band minimum of GaPAs host is modeled using perturbation theory by following Hamiltonian:

4 $${H}_{BAC}=(\begin{array}{cc}{E}_{M}(k) & {C}_{NM}\sqrt{x}\\ {C}_{NM}\sqrt{x} & {E}_{N}\end{array})$$

where *x* is the mole fraction of substitutional N atoms and *C*_*NM*_ is a constant, which describes the interaction between the nitrogen level and the conduction band. This constant depends on the semiconductor matrix and can be determined experimentally^9–11^. *E*_*M*_(*k*) is the energy dispersion of the lowest conduction band of the III-V host, which can be calculated using ***kp*** method^11^, and *E*_*N*_ is the energy of N-related states, all referenced to the top of the valence band of the III-V semiconductor host.

The interaction of dispersionless N-related states with the conduction band states leads two highly non-parabolic subbands, *E*_−_(*k*) and *E*_+_(*k*), which are given by Eq. (5):

5 $${E}_{\pm }(k)=\frac{1}{2}[{E}_{N}+{E}_{M}(k)\pm \sqrt{{[{E}_{N}-{E}_{M}(k)]}^{2}+4{C}_{NM}^{2}x}]$$

### Sample growth

100 nm-thick GaNP and GaNPAs films were grown on GaP(001) substrates by Molecular Beam Epitaxy (MBE) using a Riber Compact 21 solid source MBE system^40^. For both sets of samples, growth temperature was 450 °C (as measured by an optical pyrometer). Nitrogen has been incorporated using a valved, RF plasma source with a nominal 0.5 sccm N_2_ flow and a RF power of 400 W^41^. A V/III beam equivalent pressure ratio was set equal to or greater than 10 in order to control the incorporation of nitrogen^42,43^. In GaNPAs samples, the As flux has been varied while maintaining the P flux constant to vary the As/P ratio and to change the As content in the sample: thus leading to P nominal compositions of 95%, 90%, 70%and 60% respectively, and nominal N content of 2.5% (which is assumed to increase from one sample to the other within 0.5%: with the roughness induced by the strain relaxation)^43^.

### Optical measurements

Transmission (T) and reflection (R) spectra have been measured to determine absorption spectra. T and R measurements have been performed on a single grating 0.55-m focal-length monochromator. The optical signal has been detected by an Si *pin* photodiode using a lock-in amplifier. The absorption coefficient (α) has been calculated according to the textbook formula $\alpha (E)=-\frac{1}{d}ln(\frac{T}{{(1-R)}^{2}})$, where *d* is the thickness of the GaNP(As) film. PT and CER measurements have been performed on the same monochromator. In PT measurements the band bending inside the samples was modulated by a beam from a laser emitting at 532 nm. This beam has been modulated by a mechanical chopper at a frequency of 280 Hz. For CER measurements samples have been placed in a capacitor with the top electrode made from a copper-wire mesh which is semi-transparent for light. This electrode has been kept at a distance of ~0.5 mm from the sample surface while the sample itself was fixed to the bottom copper electrode by a silver paste. The distance between the sample surface and the top electrode has been ~0.5 mm. A maximum peak-to-peak alternating voltage of ~3.5 kV with the frequency of 285 Hz was applied. In both PT and CER measurements the sample has been illuminated by spectrum of white light from a halogen lamp (150 W) at near normal incidence^44^. The light transmitted thought (PT case) and reflected from (CER case) the sample has been dispersed through the monochromator and detected by an Si detector or a photomultiplier. Phase-sensitive detection of the PT and CER signal was performed using a lock-in amplifier. Other relevant details on CER measurements can be found in ref.^44^. For PL measurements the samples have been excited by the 405 nm line of the semiconductor laser and PL signal has been measured on a very similar but independent set-up, i.e., a single grating 0.55-m focal-length monochromator combined with the liquid nitrogen cooled Si CCD detector.
